# Comparative Interactome Profiling of Nonstructural Protein 3 Across SARS-CoV-2 Variants Emerged During the COVID-19 Pandemic

**DOI:** 10.3390/v17030447

**Published:** 2025-03-20

**Authors:** Valeria Garcia Lopez, Lars Plate

**Affiliations:** 1 Department of Biological Sciences, Vanderbilt University, Nashville, TN 37240, USA; valeria.a.garcia.lopez@vanderbilt.edu; 2 Department of Chemistry, Vanderbilt University, Nashville, TN 37240, USA; 3 Department of Pathology, Microbiology and Immunology, Vanderbilt University Medical Center, Nashville, TN 37232, USA

**Keywords:** affinity purification–mass spectrometry, virus–host interactions, variants, nsp3

## Abstract

SARS-CoV-2 virus and its variants remain a global health threat, due to their capacity for rapid evolution. Variants throughout the COVID-19 pandemic exhibited variations in virulence, impacting vaccine protection and disease severity. Investigating nonstructural protein variants is critical to understanding viral evolution and manipulation of host protein interactions. We focus on nonstructural protein 3 (nsp3), with multiple domains with different activities, including viral polyprotein cleavage, host deubiquitylation, de-ISGylation, and double-membrane vesicle formation. Using affinity purification–mass spectrometry (AP-MS), we identify differential protein interactions in nsp3 caused by mutations found in variants identified between 2019 and 2024: Alpha 20I, Beta 20H, Delta 21I, Delta 21J, Gamma 20J, Kappa 21B, Lambda 21G, Omicron 21K, and Omicron 21L. A small set of amino acid substitutions in the N-terminal region of nsp3 (nsp3.1) could be traced to increased interactions with RNA-binding proteins, which are vital in viral replication. Meanwhile, variants of the central region of nsp3 (nsp3.2) were found to share interactions with protein quality control machinery, including ER-associated degradation. In this construct, shared trends in interactor enrichment are observed between Omicron 21K and Delta 21I. These results underscore how minor mutations reshape host interactions, emphasizing the evolutionary arms race between the host and virus. We provide a roadmap to track the interaction changes driven by SARS-CoV-2 variant evolution.

## 1. Introduction

**Figure 1:**
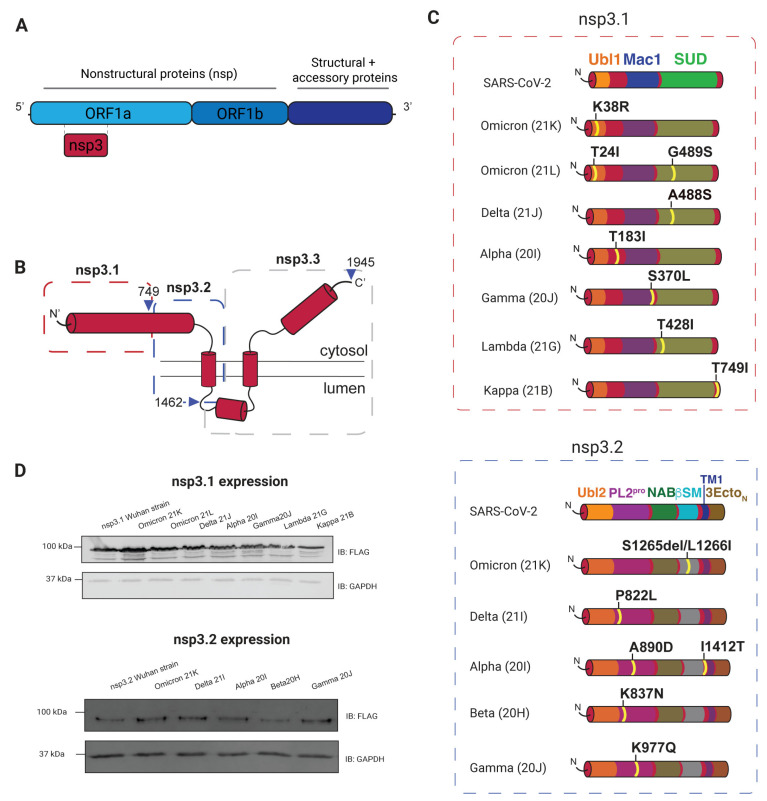
Identification and expression of SARS-CoV-2 nsp3 variants for affinity purification (AP-MS). (**A**) Schematic of SARS-CoV-2 genome with details highlighting open reading frames 1a and b, which encode nonstructural proteins. (**B**) Topological features of nsp3. Truncation sites are indicated with blue arrows and truncations used for this dataset in red and blue dashed lines. The nsp3.1 truncation (1–749) is cytosolic, while the nsp3.2 truncation (750–1462) is inserted in the ER membrane through a single-pass domain. (**C**) Domains in SARS-CoV-2 nsp3.1 and nsp3.2 truncations are mapped along with amino acid mutations for variant strains. All constructs contain a C-terminal FLAG tag for AP-MS. (**D**) Western blotting of nsp3 truncations and variants after transient transfection in HEK293T cells. GAPDH is shown as a loading control.

Severe acute respiratory syndrome coronavirus 2 (SARS-CoV-2), a member of the Coronaviridae family, is responsible for the COVID-19 pandemic. Throughout the progression of the pandemic, the SARS-CoV-2 genome has undergone mutations that have led to changes in virulence, pathogenicity, and host immune evasion [1,2,3,4,5,6]. Coronavirus genomes typically range from 26 to 32 kilobases, making them among the largest RNA virus genomes. This large genome size contributes to a high frequency of mutations and recombination events, driven by the error-prone nature of RNA-dependent RNA polymerase and frequent template switching during replication [7,8,9,10,11]. These evolutionary mechanisms enable coronaviruses to adapt to selective pressures, such as host immune defenses, while retaining functionality in the essential genes required for replication and transmission [12,13,14]. A large part of the coronavirus genome encodes open reading frames 1a and 1b (orf1a, orf1b) [15]. These open reading frames are translated into two large polyproteins, pp1a and pp1ab, which are subsequently cleaved by viral proteases into 16 nonstructural proteins (nsps) (Figure 1A). The nsps are subject to evolutionary pressures and undergo mutations as new variants arise. Changes in viral genomes subsequently lead to a change in protein–protein interactions, even between viral strains from the same families [16,17,18]. Such genomic plasticity likely allows SARS-CoV-2 to evolve mechanisms to escape host immune responses and improve transmissibility.

Affinity purification–mass spectrometry (AP-MS) interactome studies have showcased the patterns of virus and host protein interactions and can help elucidate the role of individual nsps [16,17,18,19,20,21,22,23]. Interactome profiling of SARS-CoV-2 has allowed for (1) identification of proteins that can be targeted and repurposed for anti-viral activity and (2) provides insight into the molecular evolution of pathogenic virus strains and (3) subcellular localization of interactions [18,24,25]. By mapping the host–pathogen interaction networks, interactomics studies offer a systems-level understanding of the molecular mechanisms underlying viral replication and immune evasion. These findings can contribute to developing novel therapeutic strategies by identifying key viral–host interaction nodes that can be disrupted to inhibit viral replication. Here, we focus on the interactomics of the multidomain nsp3 and nine of its variants.

Functionally, nsp3 is essential for the viral life cycle due to its self-cleaving PL2^pro^ protease domain that allows release from the viral polypeptide [26,27,28,29]. In addition to autoproteolysis, the PL2^pro^ domain is involved in de-ISGylation and deubiquitination [30,31,32,33,34]. In total, nsp3 has 10 domains, and although many domain functions remain elusive, some of these domains are essential and have a significant impact on viral replication when perturbed [35,36,37,38,39,40]. Nsp3 is pivotal in hijacking host cellular machinery to enhance viral replication efficiency.

We used AP-MS to analyze changes in nsp3 interactomics due to mutations observed in SARS-CoV-2 variants, with a specific focus on variants identified and verified by CoVariants, Nextstrain, and the World Health Organization (WHO): Omicron 21K, Omicron 21L, Lambda 21G, Kappa 21B, Delta 21I, Delta 21J, Gamma 20J, Beta 20H, and Alpha 20I [41,42,43]. We used the reference strain Wuhan-Hu-1 (Accession MN908947) and variants surveyed starting with the Alpha variant identified in late 2020; this and subsequent variants were named by the WHO according to the Greek alphabet [44,45,46,47]. The nomenclature ends with the Omicron variant that emerged in November 2021 and since then has split into various subclades (Supplementary Figure S1) [48,49,50]. We find that the Omicron nsp3 variants show the most distinct interactomes while most other interactors between variants and the Wuhan reference strain are shared. These interactome changes imply that Omicron-specific mutations could play a role in enhancing host adaptation and immune evasion. Our rich proteomics dataset provides insights into the evolution of nonstructural protein variants and the susceptibility of viral evolutionary adaptations, as these may impact infection rates and disease severity. Relatively small perturbation to protein coding sequences, like single and double point mutations, can alter interactomes and impact manipulations of host machinery.

## 2. Materials and Methods

### 2.1. Construct Design

Wuhan reference strain constructs of nsp3.1 and nsp3.2 were created and validated in previous publications and correspond to predicted SARS-CoV domains [23,51]. A previous publication from our lab has shown proper localization of fragment nsp3.1 to the cytosol and nsp3.2 localization to the ER [23]. Variant constructs were identified using Nexstrain, CoVariants, and the World Health Organization (WHO) [41,42,43]. Variant mutations identified and mapped were then mutated on the reference strain construct using a QuikChange Lightning (and Multi) Site-Directed Mutagenesis Kit (Agilent). Constructs were confirmed by sequencing.

### 2.2. Cell Culture and Transfection

HEK293T cells were grown using Dulbeccos’ Modified Eagle’s Medium (high glucose) supplemented with 10% fetal bovine serum, 1% penicillin/streptomycin, and 1% glutamine. Cell cultures were maintained at 37 °C and 5% CO_2_. Cells in 10 cm dishes were transiently transfected using calcium phosphate method with 5ug nsp3.1, nsp3.2, or GFP plasmid DNA. Medium was replenished after 16 h, and cells were harvested 24 h after that by scrapping on ice.

### 2.3. FLAG IPs

Immunoprecipitations were carried out as previously described [16,23]. Cell pellets were lysed using TNI buffer (50 mM Tris pH 7.5, 150 mM NaCl, 0.5% IGEPAL-CA-630) with “cOmplete” protease inhibitor (Roche). Lysis was performed for a minimum of 10 min on ice and a 10 min sonication in room temperature water bath. After centrifugation at 21,100× *g* for 20 min, the lysate was collected, and protein concentrations were normalized using a BCA protein assay kit (Pierce). Normalized lysates were combined with 15 µL Sepharose 4B beads (Sigma) and rocked at 4 °C for 1 h. After centrifugation at 400× *g* for 10 min, the supernatant was combined with 15 µL G1 anti-DYKDDDDK resin (GenScript) and incubated overnight at 4 °C while rocking. The resin was centrifugated at 400× *g* for 10 min and washed 4× with TNI lysis buffer. Proteins bound to the resin were eluted with 6% SDS and 62.5 mM Tris for 30 min at room temperature and 15 min at 37 °C, followed by a second elution for 15 min at 37 °C.

### 2.4. Sample Preparation for TMT Label

Five individual runs were used for analysis of nsp3.1 and nsp3.2 variant interactomes. In total, 80 IP samples were distributed across the 5 mass spectrometry runs for nsp3.1: 11× GFP (IP negative control), 11× nsp3.1 (IP Wuhan reference strain control), 8× Omicron 21K, 8× Omicron 21L, 8× Lambda 21G, 7× Kappa 21B, 7× Delta 21J, 12× Gamma 20J, and 8× Alpha 20I. While 80 IPs were performed for nsp3.2, 6 were omitted because mutation P1228L was not found to relate to a variant mutation. The IP sample replicates were as follows: 13× GFP, 13× nsp3.2, 10× Omicron 21K, 10× Delta 21I, 9× Gamma 20J, 9× Beta 20H, 10× Alpha 20I, and 6× P1228L.

Eluted proteins were precipitated with a methanol/chloroform/water (3:1:3) method and washed 3× with methanol. Pellets were air-dried before resuspending with 1% Rapigest SF (Waters). Proteins were reduced using TCEP for 30 min and alkylated with iodoacetamide for 30 min before digesting with 0.5 μg trypsin/Lys-C (Thermo, Waltham, MA, USA) overnight at 37 °C with agitation. TMTpro 16plex (Thermo) was used to label digested peptides and quenched with ammonium bicarbonate. Samples were pooled, acidified, and concentrated before removing cleaved Rapigest products by centrifugation for 45 min at 17,000× *g*.

### 2.5. MudPIT LC-MS/MS Analysis

MudPIT columns were prepared as described previously, in triphasic layers of 1.5 cm C18/SCX/C18 resins [52]. TMT-labeled samples were loaded onto the columns using a high-pressure chamber and washed with buffer A (95% water, 5% acetonitrile, 0.1% formic acid) for 30 min. Online fractionation by liquid chromatography was carried out using an Ultimate 3000 nanoLC and analyzed using an Exploris480 mass spectrometer (Thermo). Sequential injections of 10 μL were carried out for 0, 10, 20, 40, 60, 80, and 100% buffer C (500 mM ammonium acetate, 94.9% water, 5% acetonitrile, 0.1% formic acid *v*/*v*) with a final injection of 90% buffer C and 10% buffer B (99.9% acetonitrile, 0.1% formic acid *v*/*v*). Each injection had a 130 min gradient with a flow rate of 500 nl/min (0–6 min: 2% buffer B; 8 min: 5% B; 100 min: 35% B; 105 min: 65% B; 106–113 min: 85% B; 113–130 min: 2% B). ESI was performed from the tip of the microcapillary column using 2.2 kV for spray voltage, 275 °C for the ion transfer tube, and RF lens of 40%. A scan range of 400 to 1600 m/z, 120k resolution, AGC target 300%, and automatic injection times were used for the collection of MS1 spectra. MS2 spectra were gathered in a data-dependent mode using a monoisotopic peak selection: peptide, including charge state 2 to 7, TopSpeed method (3 s cycle time), isolation window 0.4 m/z, HCD fragmentation using a normalized collision energy of 32 (TMTpro), resolution 45k, AGC target of 200%, and dynamic exclusion (20 ppm window) for 60 s. Proteome Discoverer 2.4 (Thermo) was used for the identification and quantification of peptides. Fragment sequences for nsp3 were used in addition to the SwissProt human database. Searches used Sequest HT: trypsin cleavage (maximum two missed cleavages), minimum peptide length of 6 AAs, precursor mass tolerance of 20 ppm, fragment mass tolerance of 0.02 Da, dynamic modifications of Met oxidation (+15.995 Da), protein N-terminal Met loss (−131.040 Da), and protein N-terminal acetylation (+42.011 Da), static modifications of TMTpro (+304.207 Da) at Lys, and N-termini and Cys carbamidomethylation (+57.021 Da). Quantification of TMT reporter ions was performed with unique and razor peptides and excluding proteins with co-isolation interference > 25%. After the search, results were filtered to include only proteins with at least two identified peptides. Protein identification and quantification are included in Supplementary Dataset S1. Code used for data analysis can be found at https://github.com/Plate-Lab/main (Accessed on 28 February 2025).

### 2.6. Geneset Enrichment Analysis, Network Plots, and Comparative Heatmaps

Biological processes of high-confidence interactors were assessed using gene ontology (GO) enrichment analysis through Enrichr. Redundant GO terms were grouped manually based on overlapping terms, where appropriate. Network plots were generated using Cytoscape 3.10.3 [53]. Heatmaps were generated using R package pheatmap, after protein abundances were normalized to bait nsp3 levels.

## 3. Results

### 3.1. Selection and Expression of Variants

SARS-CoV-2 variants of concern were analyzed to determine variants with mutations to nsp3 that could be considered for this study. We divided nsp3 into three fragments based on previous work: nsp3.1 (amino acids 1–749), nsp3.2 (aa 750–1462), and nsp3.3 (aa 1463–1945) (Figure 1B) [23]. At the time of surveying variants, the majority of nsp3 mutations were in the nsp3.1–3.2 region, while mutations in nsp3.3 were scarce, so we excluded nsp3.3 from this study to enhance comparative power for the first two truncations. The following variants within nsp3.1 and nsp3.2 were selected, with the corresponding Pango lineages in parentheses: Omicron 21K (BA.1), Omicron 21L (BA.2), Delta 21J (no Pango lineage), Alpha 20I (B.1.1.7), Beta 20H (B.1.351), Gamma 20J (P.1), Lambda 21G(C.37), and Kappa 21B (B.1.617.1). These lineages feature eight point mutations in nsp3.1 and seven mutations in nsp3.2 (Figure 1C).

This study examines mutations across multiple domains of nsp3. In nsp3.1, they are in the ubiquitin-like domain (Ubl1), SARS-unique domain (SUD), and the macrodomain (Mac1) (Figure 1C). The Ubl1 domain has core residues that fold into a ubiquitin-like structure with disjointed helices that binds single-stranded RNA and interacts with the nucleocapsid (N) protein [54,55,56]. The Mac1 domain has been shown to combat host immune response and binds and hydrolyzes ADP-ribose and poly(ADP-ribose), which serve as a post-translational modifications [39,40,57,58,59,60]. We also observed mutations in the SUD domain, which can bind RNA, impacts viral replication, and helps with evading host immune responses [36,37,61,62].

In nsp3.2, mutations occur in the papain-like protease domain (PL2^Pro^) and betacoronavirus-specific marker (βSM) region (Figure 1C). The analysis includes five variants with six total mutations. This central fragment of nsp3 is inserted into the ER membrane and contains six domains (Figure 1C). Additionally, the transmembrane domains of nsp3.2 contribute to the stability and structural integrity of the replication organelles, mediating interactions between viral and host membranes to ensure proper compartmentalization of the replication complex. The PL2^Pro^ domain functions to cleave nsp1, nsp2, nsp3, and nsp4 from the polyprotein and has deubiquitinating and de-ISGylation activity [30,32,33,34,63]. The second region with nsp3.2 mutations, βSM, is intrinsically disordered and has an unknown function [28]. Interestingly, the domains SUD (nsp3.1) and the PL2^Pro^ (nsp3.2) have the highest frequency of mutation events, and no mutations were identified in the Mac1 domain. Four out of the five variants exhibited mutations in the papain-like protease (PL2^Pro^) domain.

All variant constructs have a C-terminal FLAG tag for immunoprecipitations. Mutations were confirmed through sequencing, and all showed comparable levels of protein expression (Figure 1D). Although a lung-derived cell line would be a more physiologically relevant model, previous studies have shown HEK293T cells as a suitable model for recapitulating viral protein interactions with host factors [16,18,23,64].

### 3.2. AP-MS of nsp3 Mutations

Host protein interactions with nsp3 were assessed by expressing the variant constructs, lysing the cells, and performing co-immunoprecipitation (IP) using anti-FLAG beads (Figure 1D). Elution samples from immunoprecipitations were reduced, alkylated, and digested with trypsin/Lys-C. Peptides were labeled using TMTpro 16plex reagents for MS2-based relative quantification. For each construct, between three and four biological replicates were pooled into a TMTpro 16plex run. Mock IPs from cells transfected with green fluorescent protein (GFP) were also included to assess the background abundance of proteins during the Co-IP process. In total, five TMTpro 16plex nsp3.1 sets were analyzed by tandem mass spectrometry with 8–12 biological replicates per variant, and five TMTpro 16plex nsp3.2 sets were analyzed with 9–10 biological replicates per variant. Proteome Discoverer 2.4 was used to identify and quantify nsp3 interactors based on the relative TMTpro reporter ion signals. High-confidence interactors were identified by using a variable cutoff method based on enrichment with a GFP background control [23] (Supplementary Figure S2).

### 3.3. Comparison of nsp3.1 Interactors

The comparative proteomics dataset for the cytosolic and N-terminal section of SARS-CoV-2 nsp3 (nsp3.1) includes seven variant constructs for a total of eight point mutations. Variant Omicron 21L contains two mutations within the fragment, and the following variants contain single mutations: Omicron 21K, Delta 21J, Alpha 21I, Gamma 20J, Lambda, and Kappa (Figure 1C).

**Figure 2:**
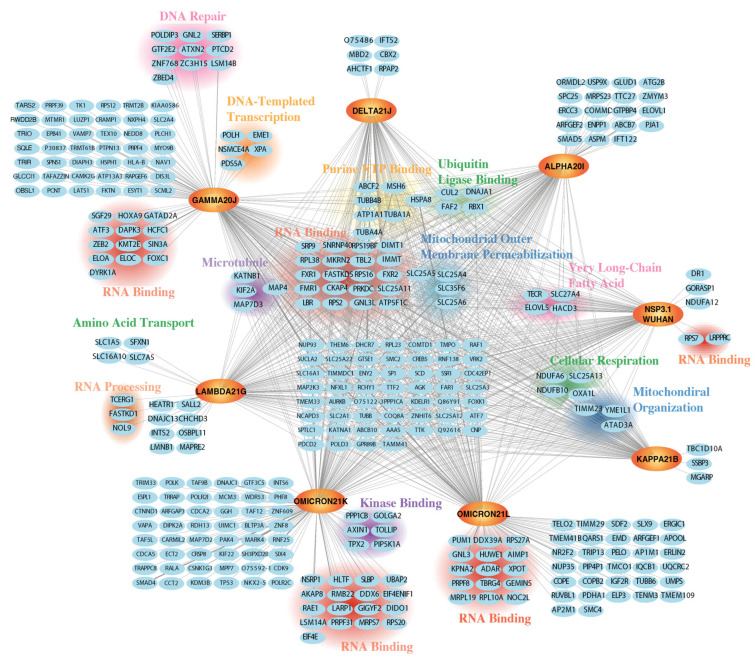
Comparative interactomics of nsp3.1 Wuhan reference strain and variants. Network plot of high-confidence host interactors that are either shared or unique to each variant. Reference strain and variant forms of nsp3.1 are shown on an orange background with bolded font. Gray lines indicate interactions between virus and host proteins and line width and share indicate highly enriched interaction compared to background. Clusters were annotated using GO term analysis and manual clustering. Omicron 21K, Omicron 21L, and Gamma 20J show the highest number of proteins that pass the filter for high-confidence interactors for those respective variants.

The nsp3.1 dataset is made up of five mass spectrometry runs. High-confidence interactors were filtered for the Wuhan reference strain nsp3.1 construct and variants by considering their fold enrichment and adjusted p-value. We identified 411 SARS-CoV-2 nsp3.1 high-confidence interactors and 300–400 interactors per variant (Supplementary Dataset S1 and S2). The interactors of the nsp3.1 reference and variant strains show mitochondrion, nucleus, and cytoplasm as the top three compartment localizations using SubcellulaRVis (Supplementary Dataset S3) [65]. We identified 104 shared protein interactors with all eight constructs (Figure 2). Gene ontology assignments through Enrichr were used to map biological processes and molecular functions represented in the list of proteins. RNA-binding proteins are the largest cluster identified in the dataset and include members of the Fragile X-related gene family (FMRPs), FMR1, FXR1, and FXR2 (Figure 2). This family of proteins is highly homologous with structural and functional similarities, only varying in the C-terminal region [66].

In addition, other shared interactors for the nsp3.1 variants clustered into a smaller subpopulation. Some of these groups include proteins involved in mitochondrial organization (AGK, TAMM41, TIMM23, YME1L1, TIMMDC1, RPL23, and ATAD3A), mitochondrial outer membrane permeabilization (SLC25A5, SLC35F6, SLC25A4, and SLC25A6), and cellular respiration (SLC25A13, NDUFA6, NDUFB10, and OXA1L). Other groupings include microtubule-associated proteins (MAP7D3, KIF2A, TUBA4A, TUBB4B, MAP4, TUBA1A, and KATNB1) and ubiquitin ligase binding (CUL2, HSPA8, RBX1, DNAJA1, and FAF2).

While there were many shared interactors, each construct had its own set of high-confidence interactors (Figure 2). Although the proteins identified are unique to the constructs, the most commonly identified grouping is RNA binding. Under this category, the Wuhan reference strain interacts with RPS7 and LRPPRC, and variant Gamma 20J interacts with DYRK1A, DAPK3, HOXA9, ATF3, SIN3A, GATAD2A, HCFC1, ELOC, KMT2E, ZEB2, ELOA, SGF29, and FOXC1. Similarly, variant Lambda 21G has three proteins (TCERG1, NOL9, and FASTKD1) categorized under RNA processing. Omicron constructs also have interactors identified as RNA-binding proteins, in total 17 proteins for Omicron 21K and 12 proteins for Omicron 21L. A few variants had high-confidence interactors that represented other categories, such as Gamma 20J with DNA-templated transcription proteins (PDS5A, EME1, POLH, NSMCE4A, and XPA), Lambda 21G with amino acid transport (SLC7A5, SLC1A5, SFXN1, and SLC16A10), and Omicron 21K with kinase-binding proteins (PIP5K1A, TPX2, GOLGA2, AXIN1, TOLLIP, and PPP1CB).

**Figure 3:**
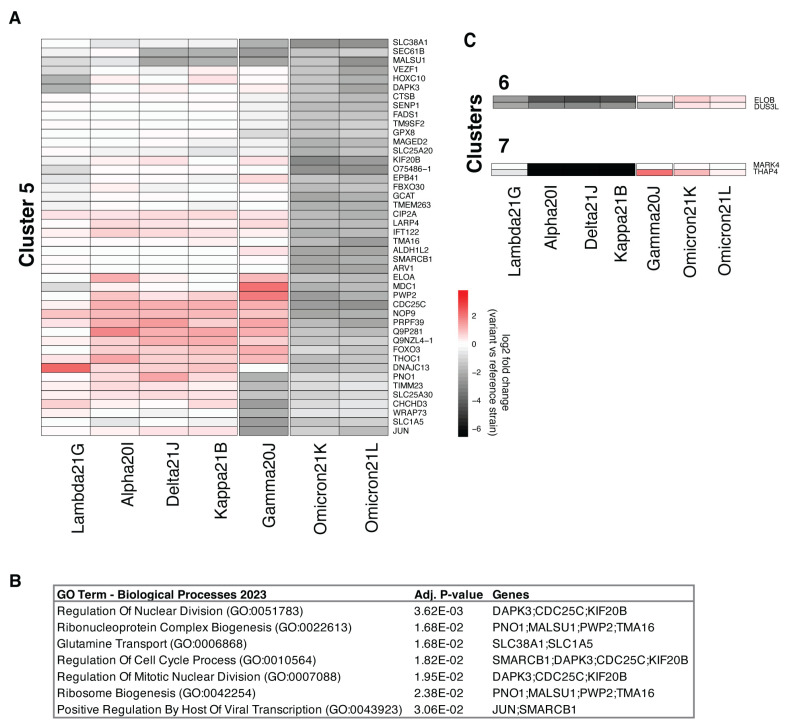
Selected clusters from variant nsp3.1 high-confidence interactors. (**A**) Heatmaps displaying the log2 fold enrichment for a given interactor comparing the Co-IP of the listed variant to the Wuhan reference strain. Red cells indicate increased log2 fold enrichment compared to the reference strain, with intensity of the color corresponding to increased enrichment. Hierarchical clustering was carried out on all proteins identified as high-confidence interactors in at least one of the constructs, using a Euclidean distance matrix supplemented with within-cluster sum of squares (WSS) and silhouette scores to determine the optimal number of clusters. Shown here is cluster 5, which is made up of proteins with increased enrichment for non-Omicron variants. (**B**) Top 7 GO term analysis results of proteins in cluster 5. (**C**) Clusters 6 and 7, made up of proteins that show increased enrichment for Omicron 21K, Omicron 21L, and Gamma 20J nsp3 variants. No GO terms were identified to be shared amongst these proteins. The full hierarchical clustering of all nsp3.1 interactors is shown in Supplementary Figure S2.

In total, 871 proteins were identified as high-confidence interactors in at least one of the constructs surveyed. The abundance levels of these proteins were assessed across all constructs, and hierarchical clustering was used to sort the proteins based on enrichment patterns (Supplementary Figure S3, Supplementary Dataset S4). Clusters were then filtered using Enrichr to identify biological processes and molecular functions identified in each cluster. Cluster 1 is the largest cluster and has a total of 474 proteins with 103 involved in RNA binding and 28 identified as being involved in gene expression (Supplementary Dataset S4). Cluster 5 is made up of 44 proteins which show enrichment in all constructs, except for Omicron 21K and Omicron 21L (Figure 3A). Of the proteins in cluster 5, three are involved in the regulation of nuclear division and four in ribonucleoprotein complex biogenesis (Figure 3B). Interestingly, clusters 6 and 7 are made up of two proteins each and were found to have increased enrichment for Omicron 21K, Omicron 21L, and Gamma 20J (Figure 3C). These four proteins are ELOB, DUS3L, MARK4, and THAP4 and have no shared GO terms.

The comparative proteomics of the N-terminal section of nsp3 (nsp3.1) resulted in 871 high-confidence protein interactors across seven constructs. A core set of 104 shared interactors was identified, with RNA-binding proteins forming the largest functional cluster. Unique interactors were identified for each variant, with the highest number found in Omicron 21K, Omicron 21L, and Gamma 20J. The number of unique interactors highlights potential variant-specific functional adaptations.

### 3.4. Comparison of nsp3.2 Interactors

**Figure 4:**
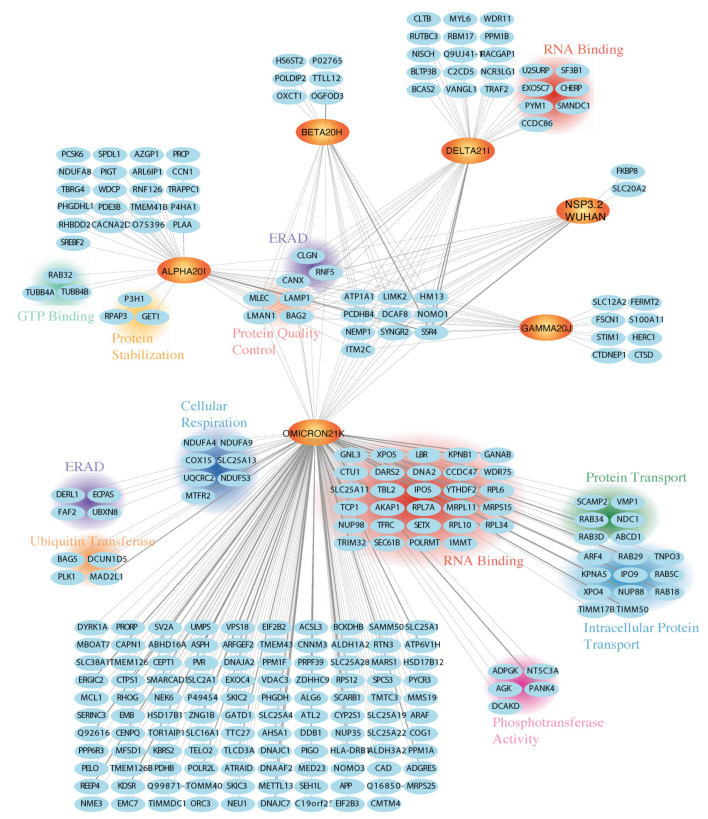
Comparative interactomics of nsp3.2 reference strain and variants. Network plot of virus–host interactors with shared high-confidence interactors in the center and high-confidence interactors unique per construct on the perimeter. Wuhan reference strain and variant forms of nsp3.2 are shown on an orange background with bolded font. Gray line intensity indicates interaction enrichment between virus and host proteins. Clusters annotate using GO term analysis and manual clustering. The Omicron 21K variant shows that the highest number of proteins pass the high-confidence interactor filter for that variant alone.

The dataset for this fragment consists of 5 mass spectrometry runs with 10 replicates each for Alpha 20I, Delta 21I, and Omicron 21K variants and 9 replicates for Beta 20H and Gamma 20J. We identified 153 high-confidence interactors for the Wuhan reference strain fragment and 100–400 high-confidence interactors for variant constructs (Supplementary Dataset S2). Using SubcellulaRVis, we identified the top two cellular compartments to be the cytosol and the ER, as expected due to the cytosolic and ER membrane-bound portion of the fragment (Supplementary Dataset S3) [65]. Notably, some proteins were identified as high-confidence interactors across all constructs in this survey of variants (Figure 4). These shared interactors include proteins related to broader protein quality control pathways, including glycosylation regulation and degradation (MLEC, LMAN1, BAG2, LAMP1, CANX, RNF5, and CLGN).

Each construct was also found to have distinct high-confidence interactors absent from other constructs (Figure 4). For instance, the Omicron 21K variant construct had more unique high-confidence interactors than other constructs. The group of interactors includes proteins involved in degradation pathways (PLK1, ECPAS, DERL1, BAG5, FAF2, UBXN8, MAD2L1, and DCUN1D5), phosphotransferase activity (DCAKD, PANK4, NT5C3A, ADPGK, and AGK), cellular respiration (UQCRC2, NDUFA4, NDUFA9, MTFR2, NDUFS3, COX15, and SLC25A13), and 22 protein transport factors. The largest subset of Omicron 21K consisted of RNA-binding proteins, with a total of 29 proteins. Similarly, this category is not unique to a single construct, as there is a subpopulation of RNA-binding proteins in the Delta 21I variant (CHERP, SF3B1, EXOSC7, PYM1, SMNDC1, U2SURP, and CCDC86). The Alpha 20I variant bound factors are involved in GTP binding (RAB32, TUBB4A, and TUBB4B) and protein stabilization (RPAP3, GET1, and P3H1).

**Figure 5:**
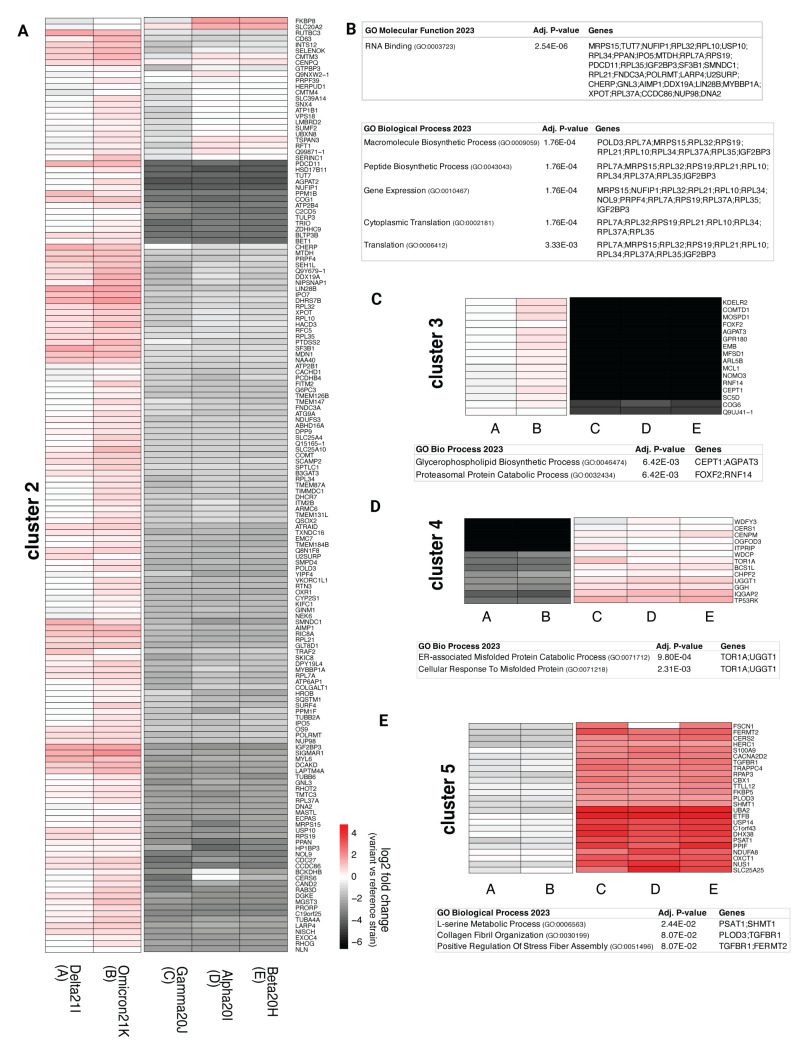
Selected clusters from variant nsp3.2 high-confidence interactors. (**A**) Selected heatmaps display the log2 fold enrichment for each interactor identified after Co-IP of a variant or reference strain. An increase in log2 fold change compared to the reference strain is indicated in red, with color intensity coordinating with enrichment for the variant construct. Clusters were determined for all proteins identified as high-confidence interactors in at least one of the constructs and using an Euclidean distance matrix. The optimal number of clusters was determined using within-cluster sum of squares (WSS) and silhouette scores. Cluster 2 shows proteins with increased enrichment for Delta 21I and Omicron 21K variants. (**B**) GO term analysis shows that many of the proteins identified in cluster 2 are RNA-binding proteins. (**C**) Cluster 3 shows more enrichment for Delta 21I and Omicron 21K compared to the other variants observed in cluster 2. Two proteins in cluster 3 were identified to be involved in glycerophospholipid biosynthesis, and two others are involved in proteasomal degradation. (**D**) Cluster 4 is made up of proteins that show increased enrichment for Gamma 20J, Alpha 20I, and Beta 20H variants. Proteins TOR1A and UGGT1 in this cluster are related to misfolded protein responses. (**E**) Cluster 5 shows mild enrichment for Delta 21I and Omicron 21K and strong enrichment for Gamma 20J, Alpha 20I, and Beta 20H. Three proteins in this cluster (PLOD3, TGFBR1, and FERMT2) are involved in either collagen or stress fibril assembly. The nsp3.2 full hierarchical clustering interactors are shown in Supplementary Figure S4.

Across all constructs, 673 high-confidence interactors were identified. These were categorized into five clusters based on enrichment patterns (Supplementary Figure S4, Supplementary Dataset S5). Cluster 2 included 314 proteins with increased enrichment in Delta 21I and Omicron 21K constructs compared to the reference strain (Figure 5A). Much like the previously discussed nsp3.1 fragment, the major subpopulation of proteins is classified as RNA binding (Figure 5B). Cluster 3 maintained interactions with the Delta 21I and Omicron 21K variants, while Gamma 20J, Alpha 20I, and Beta 20H showed severely diminished interactions compared to the Wuhan reference strain nsp3.1 (Figure 5C). Proteins in cluster 3 are involved in the glycerophospholipid biosynthesis process (CEPT1 and AGPAT3), a pathway that facilitates SARS-CoV-2 replication by enabling formation of double-membrane vesicles [67].

In contrast, clusters 4 and 5 showed increased enrichment in three constructs, excluding Omicron 21K and Delta 21I. Cluster 4 includes proteins associated with ERAD (TOR1A and UGGT1) (Figure 5D), while cluster 5 contains proteins implicated in extracellular remodeling and stress fiber assembly (PLOD3, TGFBR1, and FERMT2) (Figure 5E). Notably, extracellular matrix remodeling and stress fiber formation are critical for forming virus-loaded vesicles and have been observed in clinical COVID-19 cases and molecular models [68,69].

To summarize, the comparative proteomics analysis of nsp3.2 identified shared interactors for five variants related to protein quality control, and clustering revealed variant-specific enrichment in RNA processing, calcium transport, lipid biosynthesis, and extracellular remodeling, suggesting functional adaptations.

## 4. Discussion

Genetic variation in viral genomes has profound impacts on both viral and host processes, including viral identity and evolution, formation of new variants or strains, pathogenesis, host immune response, and drug resistance. A significant concern with SARS-CoV-2 arises from the high mutation rate of RNA viruses, which contributes to changes in pathogenicity and transmissibility [1,2,3,5]. Comparative interactomics of nsp3 variants has enabled the identification of host proteins and pathways that may be altered due to changes in the viral genome. In this work, we use quantitative proteomics to identify changes in host protein–protein interactions based on nsp3 variants: Omicron 21K and 21L, Lambda 21G, Kappa 21B, Delta 21I and J, Gamma 20J, Beta 20H, and Alpha 20I. In December of 2020, the first variant was declared and later renamed Alpha 20I, with the emergence of Gamma, Beta, and Delta subsequently following. The variants coexisted and COVID-19 infection continued at different rates depending on geographical regions and infections in subpopulations. Delta became the next dominant subclade as reports of the frequency of Alpha, Beta, and Gamma diminished. Ultimately, the Omicron variant was identified and coexisted with Delta, until it became the leading variant [47,70]. Since the time of our study, the Omicron 21L variant has given rise to new subclades, and novel variants continue to emerge [5,71,72,73]. These evolutionary adaptations can enable immune evasion and reduce vaccine efficacy, underscoring the importance of investigating variant-driven changes in virulence.

Our findings reveal that mutations in nsp3 variants tend to cluster within specific domains rather than being uniformly distributed across the protein. Notably, none of our selected variants harbored mutations in the Mac1 domain, a key region involved in counteracting the host immune response and supporting viral replication [39,40]. The absence of mutations in this domain may highlight its essential role in viral fitness. Previous studies have demonstrated that mutations and deletions of the Mac1 domain have resulted in reduced catalytic activity and viral loads and increased interferon response in both SARS-CoV-2 and murine hepatitis virus (MHV) [39,40,74]. In contrast, the PL2^pro^ domain emerged as the most frequently mutated in our analysis. As one of two proteases in the viral genome, PL2^pro^ is critical for cleaving viral polyproteins to release individual nonstructural proteins and possesses deubiquitinating and de-ISGylating activities [34]. Its essential role in viral replication and immune modulation makes it an attractive anti-viral target. Based on a published structure of the SARS-CoV-2 PL2^pro^ domain, the catalytic region does not seem to be impacted by variant mutations, which may be indicative of viral protein immune evasion in enzymatic regions (Supplementary Figure S5) [75]. Previous studies have investigated the impact of nonstructural protein variants on phosphorylation, yet it would be beneficial to expand this into variant changes in ISGylation and ubiquitination to assess pro- or anti-viral changes [64].

Fragment nsp3.1, which lacks a transmembrane region, localizes to the cytosol, while nsp3.2 is inserted into the ER membrane. The differences in localization allow us to validate some of our findings; for example, we identify cytosolic proteins with the enrichment of nsp3.1, whereas enrichment on nsp3.2 results in ER-related proteins involved in protein quality control (Supplementary Dataset S3).

Consistent with previous findings that identified RNA-binding proteins as interactors of nsp3 in SARS-CoV-2 and less pathogenic coronaviruses, our results further indicate that variant nsp3 constructs exhibit an increased abundance of RNA-binding proteins compared to Wuhan reference strain SARS-CoV-2 [75]. Given the central role of RNA in the SARS-CoV-2 life cycle, both as the viral genome and through its interaction with host processes, RNA-binding proteins likely contribute to viral replication, transcription, immune evasion, and host pathway modulation [75,76]. A previous study has shown that RNA transcripts and proteins have the strongest response to variant infection compared to the reference strain [64]. The role of nsp3 for the formation of double-membrane vesicles serves as a protective mechanism for viral replication. Additionally, viruses may generate membrane-less compartments through liquid–liquid phase separation as protective compartments for viral replication. RNA-binding proteins are key players in the formation of phase-separated membrane-less organelles, including in viral infection settings [77,78,79]. Existing data suggest that interactions between the nucleocapsid (N) protein and nsp3 can regulate separation [80,81]. This may be indicative of the importance of host RNA-binding proteins in helping mediate the viral-driven hijacking of the cell, and the increased abundance of RNA-binding proteins in nsp3 variants may suggest an increased dependence on biomolecular condensates.

We found prominent interactions between nsp3.1 and members of the Fragile X-related protein family (FMRPs), FMR1, FXR1, and FXR2. In recent work, it was found that there is a direct interaction between the FMRPs and nsp3 through a peptide motif [82]. Disruption of the interaction between FMRPs and nsp3 leads to reduced viral replication, making the interaction between these proteins pro-viral. The interaction between nsp3 and FMRPs has been observed in previous studies of the nsp3 Wuhan reference strain and some variants, yet here we show that this interaction occurs with a wide spectrum of variant forms of nsp3 [23,64,83]. The identification of FMRPs as high-confidence interactors in all constructs of nsp3 can serve as supporting evidence of the importance of this interaction for viral replication. The FMRPs are part of the RNA-binding protein group that we identified in our interactions. In the future, we aim to assess if other interactors found in our dataset could be implicated in the formation of stress granules, a type of membrane-less organelle. We are particularly interested in investigating if interactions with DIMT1, a methyltransferase, are conserved during viral infection and in biologically relevant cell lines. DIMT1 is part of the intracellular membrane-less organelle gene ontology category, which includes FMRPs, but has yet to be directly implicated in stress granule formation during viral infection. DIMT1 is an intriguing hit because another methyltransferase, protein arginine methyltransferase 1 (PRMT1), has been identified within stress granules and shown to promote stress granule assembly [84].

Within the nsp3.2 dataset, we identified malectin (MLEC) as a high-confidence interactor for the Wuhan reference strain and its variants. Previous proteomics studies have implicated MLEC as an interactor of SARS-CoV-2 nonstructural proteins, specifically nsp2 [16,85]. The glycoprotein quality control factor, MLEC, may be implicated in increased replication for SARS-CoV-2 and murine hepatitis virus (MHV), a well-established infection model [85].

Clinical observations have linked Delta variant infections to increased disease severity and prolonged viral shedding, while Omicron infections are associated with milder disease but higher viral shedding compared to Delta [47,50,70]. Our findings show notable differences between Omicron and Delta variant interactors that are most drastic in the nsp3.1 dataset. While nsp3.1 interactomes for Omicron and Delta diverged significantly, nsp3.2 variants showed similar interaction patterns, clustering Omicron 21K and Delta 21I. This indicates that Omicron and Delta variant mutations in nsp3.1 led to a divergence in interaction patterns, whereas the interactomes with nsp3.2 variants are similar. Although our study provides valuable insights, it faces certain limitations. The lack of biological context, the remodeling that occurs during viral infection, and the use of the HEK293T cell line as a model (rather than the primary epithelial lung cells that are the main infection site for SARS-CoV-2) mean that not all the interactions identified in this study may be directly relevant to the viral infection process. This highlights the importance of studying nsp3 in its full form and during viral infection, as it allows for a more comprehensive aggregation of data, which can subsequently facilitate more accurate conclusions regarding the predominant interactions [86].

In total, we investigated the protein interactome across nsp3 variants, specifically focusing on the fragments nsp3.1 and nsp3.2. Our dataset represents the most variant-diverse nsp3 interactome to date with a total of 9 variants and 15 mutations. The study of nonstructural protein variants offers critical insights into their functional diversity, evolutionary adaptations, and roles in pathogenicity. By elucidating key structure–function relationships, mechanisms of drug resistance, and host–pathogen interactions, these findings provide a foundation for developing broad-spectrum therapeutics and improving our understanding of disease progression at a molecular level. These findings can inform the development of broad-spectrum therapeutics and enhance our ability to predict disease outcomes and design effective interventions. The comparative analysis provided here can also be utilized to gain insight into possible functions of domains of nsp3 that remain unknown. By comparing the variants with mutations in similar regions, we can gain insight into interactors that are conserved or altered due to genetic changes. The Omicron 21L variant continues to evolve and generate sublineages that dominate SARS-CoV-2 evolution, as opposed to the emergence of new variants (Supplementary Figure S1). This led the WHO to supplement the variants of concern (VOCs) list to include VOC lineages under monitoring (VOC-LUM) [42]. These new sublineages maintain the mutations found in their ancestor and have additional mutations that can impact viral properties [87]. All Omicron VOC-LUM have ancestral Omicron 21L mutations, and 16 out of 17 have additional mutations in nsp3 [41]. Future interactome studies of viral variants would continue to increase our understanding of mutations that lead to divergent molecular mechanisms or have an impact on virulence, particularly in an infection setting.

## Data Availability

The mass spectrometry proteomics data have been made accessible through the ProteomeXchange Consortium via the PRIDE partner repository. Dataset identifier: PXD061195. Other data are contained within this manuscript and Supplementary Materials.

## Supplementary Materials

The following supporting information can be downloaded at: https://www.mdpi.com/article/10.3390/v17030447/s1, Figures S1–S5; Dataset S1–S5. Figure S1. Phylogenetic Tree of SARS-CoV-2 Clades. This scheme is modified from Covariants and shows the phylogenetic relationships of SARS-CoV-2 variants [41]. The emergence of new clades (rows) and subclades (in line with ancestral clade). Variants included in our study are highlighted with color circles and names bolded in red. Figure S2. TMT Intensity Distribution. (A) Raw abundance of all mass spectrometry runs, grouped by nsp3.1 construct. (B) Normalized TMT intensity of nsp3.1 constructs, plotted on log10 TMT scale. (C) Raw TMT abundance of all nsp3.2 mass spectrometry runs. (D) Normalized and log10 transformed TMT intensity of nsp3.2 constructs. Normalized values used for subsequent filtering. Figure S3. Comparative heatmap of nsp3.1 variant interactors. Heatmap of proteins identified in at least one of the variant or reference strain Co-IPs for nsp3.1. Color intensity corresponds to enrichment intensity, with red showing increased enrichment of a protein compared to the level detected in Co-IP of the Wuhan reference strain. Hierarchical clustered heatmap of high confidence interactors identified for at least one nsp3.1 variant or the Wuhan reference strain construct. Clustering done using Euclidean distance matrix. Seven unique clusters identified. Variant clustering resulted in 3 clusters: (1) Lambda 21G, Alpha 20I, Delta 21J, Kappa 21B (2) Gamma20J (3) Omicron 21K and Omicron 21L. Figure S4. Comparative heatmap of nsp3.2 variant interactors. Heatmap of nsp3.2 proteins identified as high confidence interactor in at least one of the Co-IPs, either variant or reference strains. Enrichment is represented with color intensity, red shows an increased enrichment compared to that detected in Co-IP of the Wuhan reference strain. Hierarchical clustering was done after normalization to Wuhan reference strain signal. Five unique clusters identified for proteins. Variant clustering resulted in 2 clusters: (1) Delta 21I, Omicron 21K (2) Gamma 20J, Alpha 20I, and Beta 20H. Figure S5. Location of variant mutations in the nsp3.2 PL2^pro^ domain structure. (A) PL2^pro^ domain structure (PDB: 6WZU) [33] of SARS-CoV-2. Mutation sites are highlighted in green circles and labeled with variant name, while the catalytic triad highlighted in red. Supplementary Dataset S1. Protein identification and quantification of nsp3.1 and nsp3.2 reference strain and variant strains affinity purification. Supplementary Dataset S2. TMT channel organization for each quantitative proteomics experiment. Supplementary Dataset S3. List of subcellular localization results from SubcellulaRVis and EnrichR of high confidence shared interactors amongst all constructs and SubcellulaRVis results for the individual Wuhan references and variant high confidence interactors. Supplementary Dataset S4. List of high confidence interactors identified in the nsp3.1 fragment experiments along with results from Enrichr searches. Supplementary Dataset S5. List of high confidence interactors identified for the nsp3.2 fragment affinity purification experiments, including results from Enrichr searches.

## Abbreviations

The following abbreviations are used in this manuscript:

Nsp3	Nonstructural protein 3
AP-MS	Affinity purification–mass spectrometry
IP	Immunoprecipitation
WHO	World Health Organization
TMTs	Tandem mass tags
Ubl1	Ubiquitin-like domain
SUD	SARS-unique domain
Mac1	Macrodomain
N protein	Nucleocapsid protein
PL2^Pro^	Papain-like Protease
βSM	Betacoronavirus-specific marker
FMRP	Fragile X-related protein
GO	Gene ontology
ERAD	Endoplasmic reticulum-associated degradation
MHV	Murine hepatitis virus
VOCs	Variants of concern
VOC-LUM	Variants of concern lineages under monitoring
