# Supplementary material for: Comparative Interactome Profiling of Nonstructural Protein 3 Across SARS-CoV-2 Variants Emerged During the COVID-19 Pandemic
Source: Viruses. 2025 Mar 20;17(3):447. doi: 10.3390/v17030447 (PMC11946765; doi:10.3390/v17030447)
Supplement: Supplementary file 1 [file viruses-17-00447-s001.zip › Supplementary_Viruses_v3_final.pdf]

Supplementary Information for

Comparative Interactome Profiling of Nonstructural Protein 3 Across SARS-CoV-2 Variants Emerged During the COVID-19 Pandemic

Valeria Garcia Lopez[1], Lars Plate [1-3,*]

1 Department of Biological Sciences, Vanderbilt University, Nashville, TN 37240, USA.
2 Department of Chemistry, Vanderbilt University, Nashville, TN 37240, USA.
3 Department of Pathology, Microbiology and Immunology (PMI), Vanderbilt University Medical Center, Nashville, TN 37232, USA.

* Correspondence: lars.plate@vanderbilt.edu;
Departments of Biological Sciences, Chemistry, and PMI
Vanderbilt University
Nashville, TN 37340
Phone: (615)-343-3405

TABLE OF CONTENT

Supplementary Figures S1-S5…….ppS2-S9

Supplementary Datasets ………… pS10

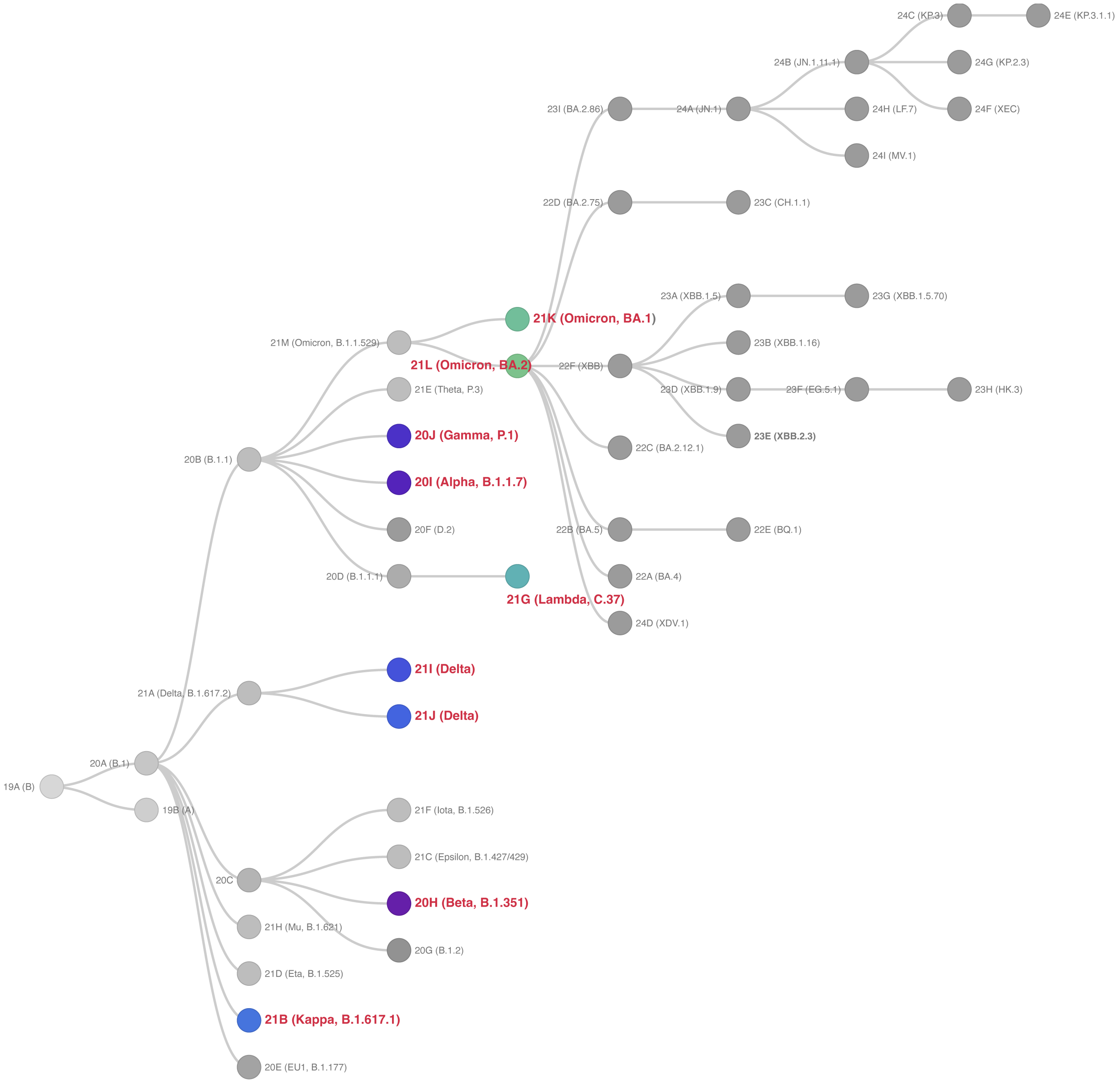

Supplementary Figure S1. Phylogenetic Tree of SARS-CoV-2 Clades. This scheme is modified from Covariants and shows the phylogentic relationships of SARS-CoV-2 variants[41]. The emergence of new clades (rows) and subclades (in line with ancestral clade). Variants included in our study are highlighted with color circles and names bolded in red.

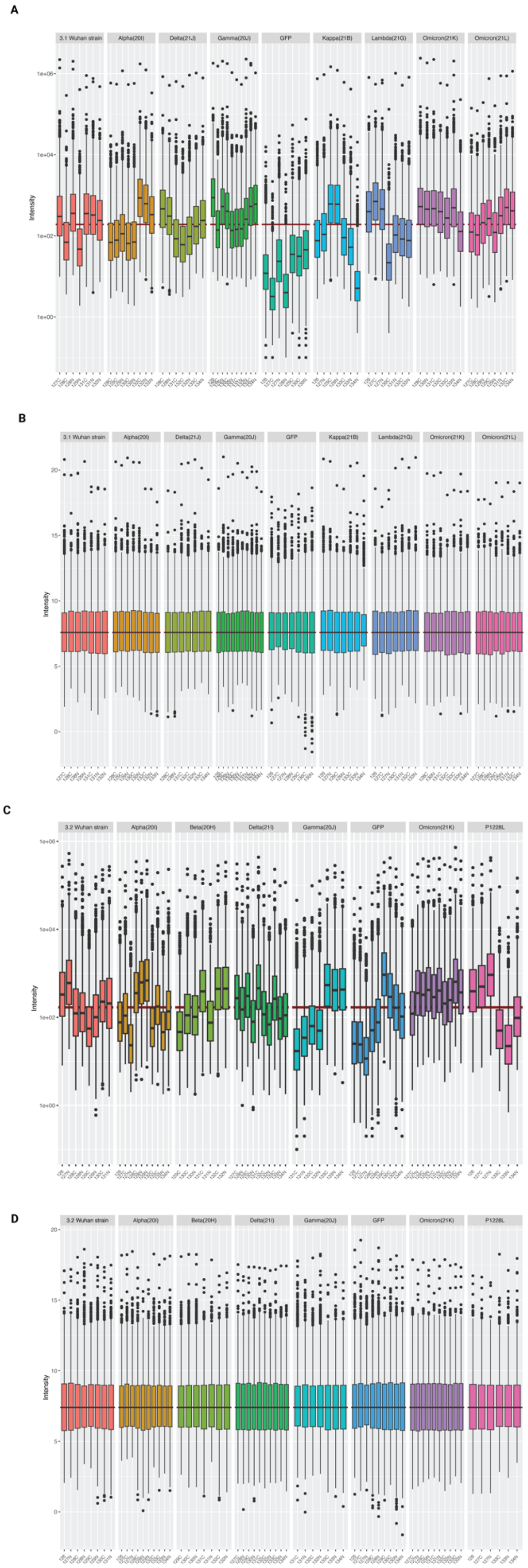
A
3.1 Wuhan strain
Alpha(20I)
Delta(21J)
Gamma(20J)
GFP
Kappa(21B)
Lambda(21G)
Omicron(21K)
Omicron(21L)
Intensity
1e+06
1e+04
1e+02
1e+00
B
3.1 Wuhan strain
Alpha(20I)
Delta(21J)
Gamma(20J)
GFP
Kappa(21B)
Lambda(21G)
Omicron(21K)
Omicron(21L)
Intensity
20
15
10
5
0
C
3.2 Wuhan strain
Alpha(20I)
Beta(20H)
Delta(21I)
Gamma(20J)
GFP
Omicron(21K)
P1228L
Intensity
1e+06
1e+04
1e+02
1e+00
D
3.2 Wuhan strain
Alpha(20I)
Beta(20H)
Delta(21I)
Gamma(20J)
GFP
Omicron(21K)
P1228L
Intensity
20
15
10
5
0

Supp. Figure S2. TMT Intensity Distribution. A) Raw abundance of all mass spectrometry runs, grouped by nsp3.1 construct. B) Normalized TMT intensity of nsp3.1 constructs, plotted on log10 TMT scale. C) Raw TMT abundance of all nsp3.2 mass spectrometry runs. D) Normalized and log10 transformed TMT intensity of nsp3.2 constructs. Normalized values used for subsequent filtering.

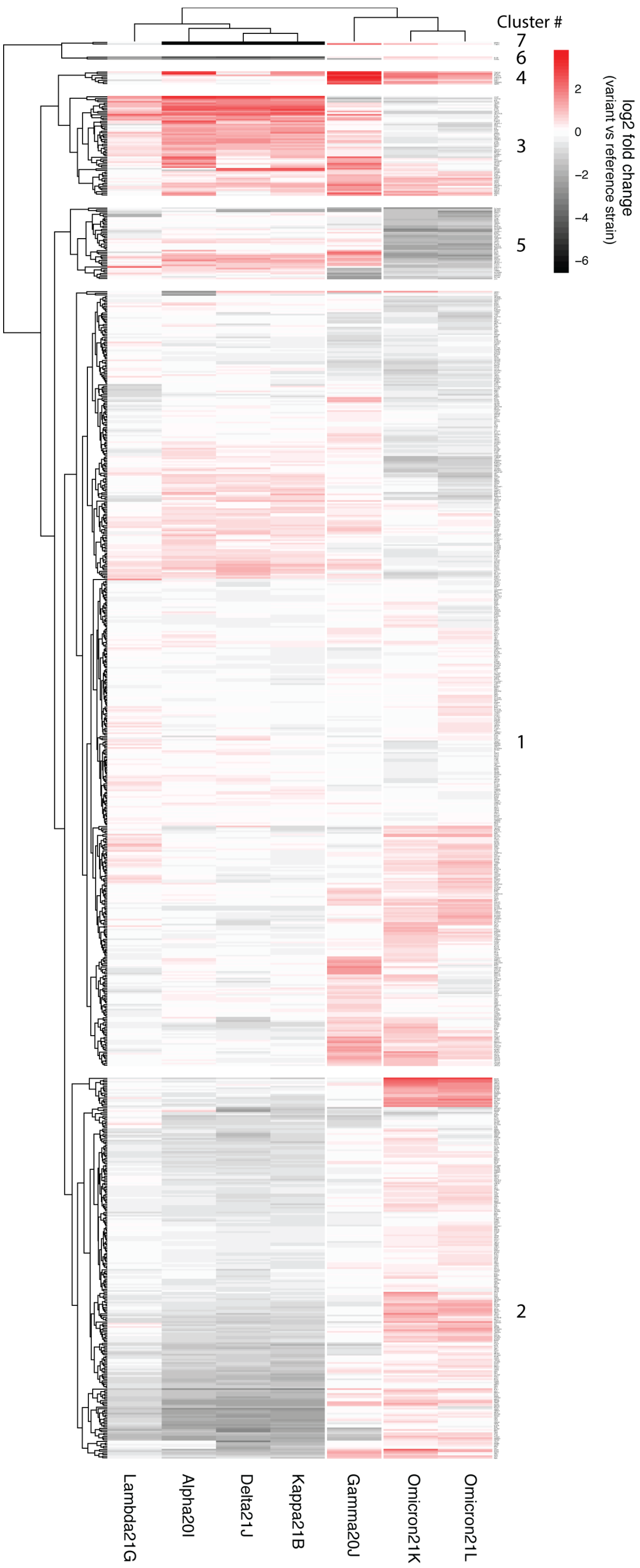
Cluster #
7
6
4
3
5
1
2
log2 fold change
(variant vs reference strain)
2
0
−2
−4
−6
Lambda21G
Alpha20I
Delta21J
Kappa21B
Gamma20J
Omicron21K
Omicron21L

Supp. Figure S3. Comparative heatmap of nsp3.1 variant interactors.

Heatmap of proteins identified in at least one of the variant or reference strain Co-Ips for nsp3.1. Color intensity corresponds to enrichment intensity, with red showing increased enrichment of a protein compared to the level detected in Co-IP of the Wuhan reference strain. Hierarchical clustered heatmap of high confidence interactors identified for at least one nsp3.1 variant or the Wuhan reference strain construct. Clustering done using Euclidean distance matrix. Seven unique clusters identified. Variant clustering resulted in 3 clusters: 1) Lambda 21G, Alpha 20I, Delta 21J, Kappa 21B 2) Gamma20J 3) Omicron 21K and Omicron 21L.

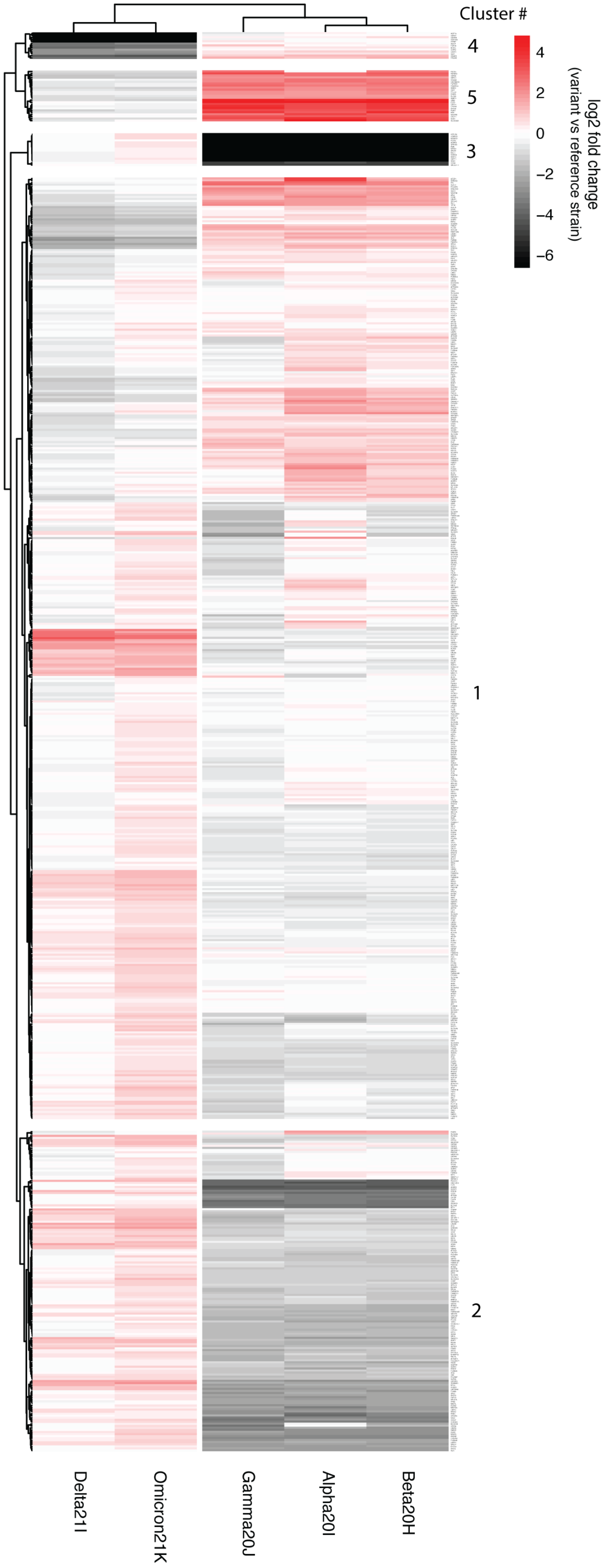
Cluster #
4
5
3
1
2
log2 fold change
(variant vs reference strain)
4
2
0
−2
−4
−6
Delta21I
Omicron21K
Gamma20J
Alpha20I
Beta20H

Supp. Figure S4. Comparative heatmap of nsp3.2 variant interactors.
Heatmap of nsp3.2 proteins identified as high confidence interactor in at least one of the Co-Ips, either variant or reference strains. Enrichment is represented with color intensity, red shows an increased enrichment compared to that detected in Co-IP of the Wuhan reference strain. Hierarchical clustering was done after normalization to Wuhan reference strain signal. Five unique clusters identified for proteins. Variant clustering resulted in 2 clusters: 1) Delta 21I, Omicron 21K 2) Gamma 20J, Alpha 20I, and Beta 20H.

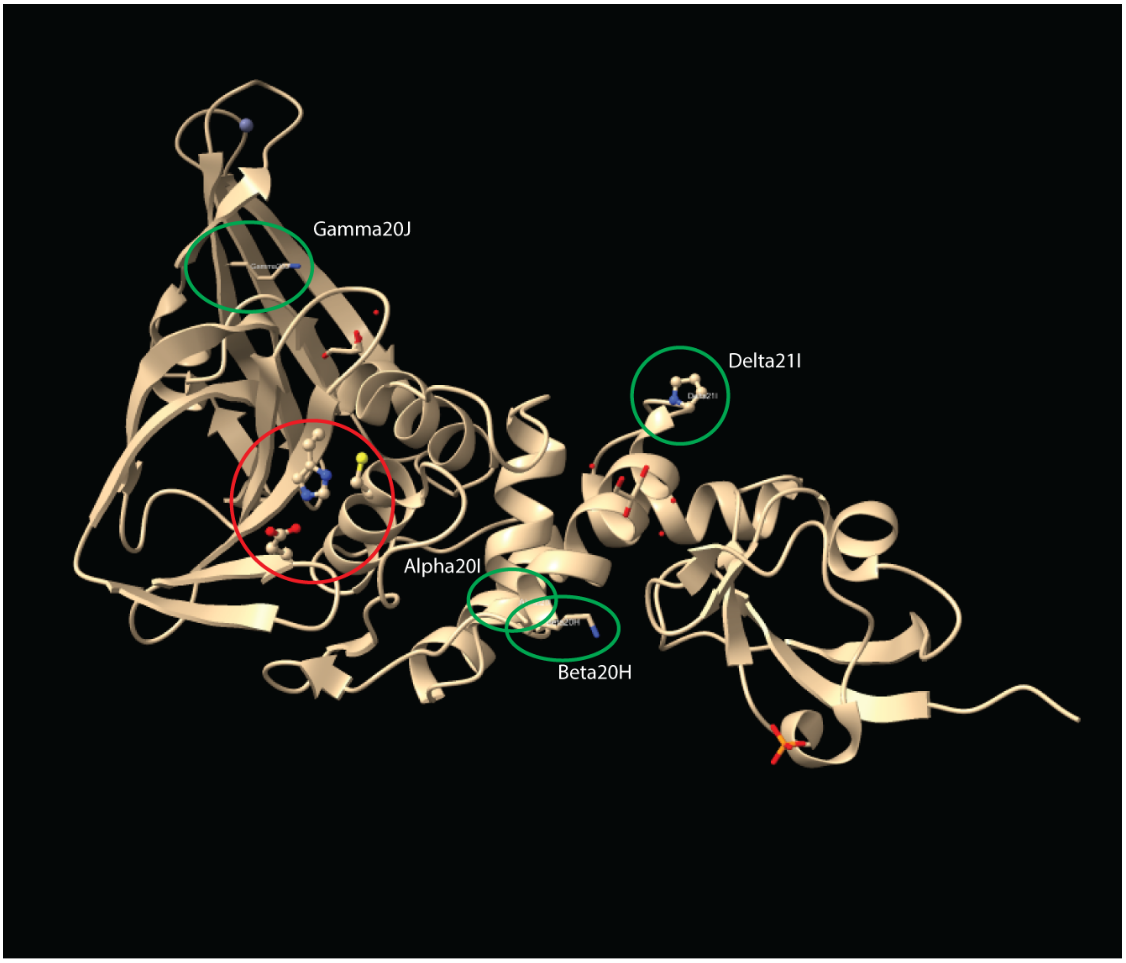

Supp. Figure S5. Location of variant mutations in the nsp3.2 PL2^{pro} domain structure. A) PL2^{pro} domain structure (PDB: 6WZU[33]) of SARS-CoV-2. Mutations sites are highlighted in green circles and labeled with variant name, while the catalytic triad highlighted in red.

Supplementary Datasets

(supporting datasets available as Excel files)

Supplementary Dataset S1. Protein identification and quantification of nsp3.1 and nsp3.2 reference strain and variant strains affinity purification.

Supplementary Dataset S2. TMT channel organization for each quantitative proteomics experiment.

Supplementary Dataset S3. List of subcellular localization results from SubcellulaRVis and EnrichR of high confidence shared interactors amongst all constructs and SubcellulaRVis results for the individual Wuhan references and variant high confidence interactors.

Supplementary Dataset S4. List of high confidence interactors identified in the nsp3.1 fragment experiments along with results from Enrichr searches.

Supplementary Dataset S5. List of high confidence interactors identified for the nsp3.2 fragment affinity purification experiments, including results from Enrichr searches.
